# The superior fault tolerance of artificial neural network training with a fault/noise injection-based genetic algorithm

**DOI:** 10.1007/s13238-016-0302-5

**Published:** 2016-08-09

**Authors:** Feng Su, Peijiang Yuan, Yangzhen Wang, Chen Zhang

**Affiliations:** 1 Robotics Institute, Beihang University, Beijing, 100191 China; 2 State Key Laboratory of Membrane Biology, School of Life Sciences, Beijing, 100871 China; 3 PKU-IDG/McGovern Institute for Brain Research, Peking University, Beijing, 100871 China

**Keywords:** artificial neural networks, fault tolerance, genetic algorithm

## Abstract

Artificial neural networks (ANNs) are powerful computational tools that are designed to replicate the human brain and adopted to solve a variety of problems in many different fields. Fault tolerance (FT), an important property of ANNs, ensures their reliability when significant portions of a network are lost. In this paper, a fault/noise injection-based (FIB) genetic algorithm (GA) is proposed to construct fault-tolerant ANNs. The FT performance of an FIB-GA was compared with that of a common genetic algorithm, the back-propagation algorithm, and the modification of weights algorithm. The FIB-GA showed a slower fitting speed when solving the exclusive OR (XOR) problem and the overlapping classification problem, but it significantly reduced the errors in cases of single or multiple faults in ANN weights or nodes. Further analysis revealed that the fit weights showed no correlation with the fitting errors in the ANNs constructed with the FIB-GA, suggesting a relatively even distribution of the various fitting parameters. In contrast, the output weights in the training of ANNs implemented with the use the other three algorithms demonstrated a positive correlation with the errors. Our findings therefore indicate that a combination of the fault/noise injection-based method and a GA is capable of introducing FT to ANNs and imply that the distributed ANNs demonstrate superior FT performance.

**Electronic supplementary material:**

The online version of this article (doi:10.1007/s13238-016-0302-5) contains supplementary material, which is available to authorized users.

## Introduction

The brain is composed of biological neural networks (BNNs) that contain billions of interconnecting neurons with the ability to perform computations. Artificial neural networks (ANNs), mathematical models that mimic BNNs, are typically built as structured node groups with activation functions and connection weights that are adjusted based on the applied learning rules (Hampson, 1991, 1994; Basheer and Hajmeer, 2000; Krogh, 2008). Because of their powerful computational and learning abilities, ANNs are being used increasingly in various fields, including computation, engineering, machine learning, clinical medicine, and cognitive science (Presnell and Cohen, 1993; Baxt, 1995; Dybowski and Gant, 1995; Forsstrom and Dalton, 1995; Kamimura et al., 1996; Almeida, 2002; Lisboa, 2002; Rajan and Tolley, 2005; Lisboa and Taktak, 2006; Patel and Goyal, 2007; Hu et al., 2013; Street et al., 2013; Azimi et al., 2015).

Fault tolerance (FT), an important feature of BNNs, ensures the fidelity and reality of a system’s input-output relationship. The FT of BNNs is thought to rely on extensive parallel interconnections, distributed information storage and processing, and self-learning and self-organizing characteristics. For instance, Alzheimer’s patients lose a significant number of neurons (sometimes equaling half the normal brain mass) but still maintain certain brain functions (Fayed et al., 2012; Li et al., 2012; Weiner et al., 2015; Pini et al., 2016). Moreover, structural measurements of various areas of the brain have revealed that brain volume has no direct correlation with cognitive decline in patients (Braskie and Thompson, 2014). Fault tolerance is also an important consideration in the construction of ANNs, especially in highly variable or “fail-safe” systems (Protzel et al., 1993; Phatak and Koren, 1995b). A fault-tolerant ANN is a special ANN system designed to work normally, or at least to a certain degree of normalcy, even if some of its components are unexpectedly damaged. Recently, FT performance has become more important, partly due to the fact that the soft errors caused by transient faults are an unavoidable concern in very large-scale integration (VLSI) technology, whose dimension is approaching the nanoscale (Mahdiani et al., 2012).

To construct a fault-tolerant ANN, neurons (nodes) are replicated in the hidden layer (Emmerson and Damper, 1993; Medler and Dawson, 1994; Phatak and Koren, 1995a; Tchernev et al., 2005). In this way, FT is introduced to the ANNs at the expense of increased complexity. For instance, ANNs with thousands of artificial neurons and up to a million interconnections in the hidden layer are required to solve complex problems, such as mapping landslide susceptibility (Arnone et al., 2014), modeling pectus excavatum corrective prostheses (Rodrigues et al., 2014), and reconstructing traffic networks (Jiang et al., 2014). However, this increase in network complexity makes the hardware implementation of ANNs relatively difficult and inefficient. Other methods that have been proposed to build fault-tolerant ANNs include an adjustment of the distributing weight values (Cavalieri and Mirabella, 1999a) using an empirical equation to deduce the mean prediction error (Sum and Leung, 2008) and adopting two objective functions (i.e., one that deals with open-weight fault and another that deals with open node fault (Mak et al., 2011)) during the training process to improve network FT. However, to our knowledge, no studies have investigated in detail whether a genetic algorithm might enhance the FT of ANNs.

A genetic algorithm (GA) is a heuristic algorithm used to search for a non-random optimal solution to a problem by mimicking the evolutionary process of natural selection (Holland, 1975; Goldberg, 1989). The GA process is iterative and includes initialization, selection, and genetic operation. The genetic operation usually consists of inheritance, mutation, and crossover. In each iteration, which is also called a generation, a fitness function is used to evaluate the fitness of individuals to find the best solution. Thus, a GA requires only a solvable function, which makes it suitable for complex and non-linear problems. Genetic algorithms have been applied to solve a variety of problems, especially when the basic functions are not discontinuous or non-differentiable (Forrest, 1993; Maddox, 1995; Willett, 1995; Pedersen and Moult, 1996; Meurice et al., 1998; Weber, 1998; Liu and Wang, 2001; Rothlauf et al., 2002; Jamshidi, 2003; Leardi, 2007; Wu, 2007; Gerlee et al., 2011; Manning et al., 2013; Pena-Malavera et al., 2014).

Thus, this study proposes an approach that combines an FIB learning algorithm with a GA to build fault-tolerant ANNs and to demonstrate this method’s superior FT performance in comparison with that of a general GA (GE-GA) and two classic existing algorithms, the back-propagation (BP) algorithm and the modification of weight (MW) methods, in solving an exclusive OR (XOR) problem or an overlapping classification problem.

## Results

### Training ANNs to solve an XOR problem with a GA

**Figure 1:**
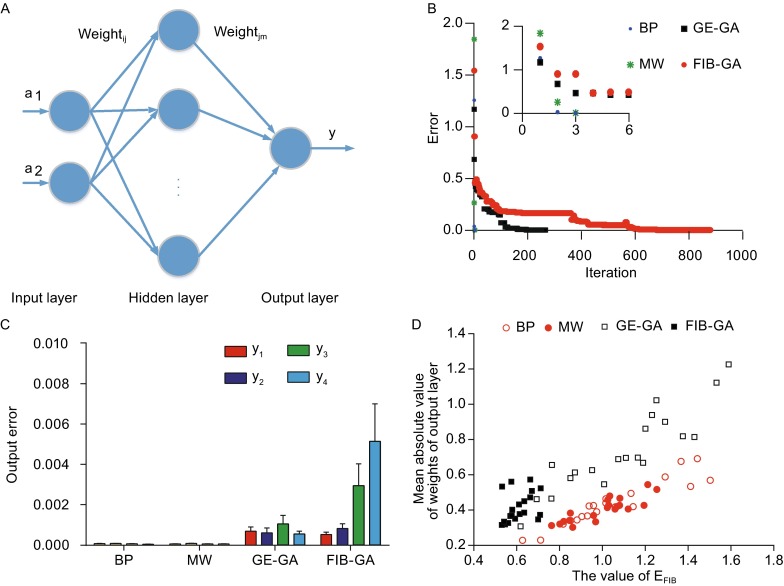
**The use of BP, MW, GE-GA, and FIB-GA in training ANNs to solve an XOR problem**. (A) The topology of the artificial neural networks. (B) The plot of the fitting errors versus the number of iterations. The inset shows the changes of the error within the initial six iterations. (C) The summary graph comparing the error of each element in the output vectors between BP ANN, MW ANN, GE-GA ANN, and FIB-GA ANN. (D) The plot of the weights for the output neuron versus the fitting errors in ANN training with the BP, MW, GE-GA, and FIB-GA

An XOR problem was used to train the ANN with either a GE-GA or an FIB-GA. Figure 1A illustrates the architecture of the ANN, and Fig. S1 illustrates the artificial neuron model. Two classic algorithms were included in the comparisons: the back-propagation (BP) and the modification of weights (MW). The BP algorithm is a traditional learning method based on a gradient descent, and the MW algorithm modifies the weight during the learning phase if the absolute value of the weight exceeds a certain threshold. For each experiment, the training proceeded until the terminating condition (i.e., error less than 0.001 or number of iterations reaching 1,000) was satisfied. Figure 1B illustrates changes in the error (or minimum error in training with a GE-GA or FIB-GA) in one iteration versus the number of iterations. Errors with all four fitting methods declined with increasing iterations. The BP and MW methods reached the terminating condition much faster (BP: 3.0 ± 0.0, MW: 3.8 ± 0.8; GA: 610.6 ± 274.8; FT: 905.6 ± 148.4 iterations) compared with the other two methods. Furthermore, the ${\text{Error}}_{\text{BP}}$, ${\text{Error}}_{\text{MW}}$ and ${\text{Error}}_{{{\text{GE}} - {\text{GA}}}}$ decayed approximately in an exponential manner (τ = 5.6196 ± 0.8967, fitting function: ${\text{f}}({\text{x}}) = {\text{a}} \cdot {\text{e}}^{{ - \tau {\text{x}}}}$, 4.1171 ± 0.6494, and 0.0186 ± 0.0050 iteration^−1^, respectively); however, the ${\text{Error}}_{{{\text{FIB}} - {\text{GA}}}}$ decayed much more slowly and in an irregular manner (Fig. 1B), suggesting low efficiency in the parameter optimization process. As there are four elements in the output vector ($${\text{c}} = \left[ {\begin{array}{*{20}c} {{\text{c}}_{ 1} }\, & {{\text{c}}_{ 2} }\, & {{\text{c}}_{ 3} }\, & {{\text{c}}_{ 4} } \\ \end{array} } \right]$$) and the calculated error in one iteration comprises the average from all four individual elements, we also examined, in each training period, the error of each element that is given by

$${\text{Error}}_{-}{\text{c}}_{\text{i}} = {\text{c}}_{\text{i}}^{\text{calculated}} - {\text{c}}_{\text{i}}^{\text{actual}} \quad\left( {{\text{i }} = { 1},{ 2},{ 3},{ 4}} \right).$$

Figure 1C illustrates the fluctuations in the error of each element during 20 independent trainings. The average fluctuations during the training of ANNs with BP, MW, GE-GA, and FIB-GA were BP: 0.0001 ± 0.0000, MW: 0.0001 ± 0.0000, GE-GA: 0.0007 ± 0.0009, and FIB-GA: 0.0024 ± 0.0025, respectively. Statistical analysis revealed that the FIB-GA method showed the biggest fluctuation when compared with the other three methods (Table S1). Taken together, all four methods demonstrated the capability of training the ANN successfully, although at different speeds.

Typically, in an FT ANN, the impact of each node is distributed as evenly as possible so as to avoid *dominant* nodes. Thus, we evaluated the correlation index between different ANN parameters and the fitting errors. All 25 parameters were grouped into four categories: 12 weights (${\text{weight}}_{\text{ij}}$) and six biases (${\text{bias}}_{\text{j}}$) for neurons in the hidden layer and six weights (${\text{weight}}_{\text{jm}}$) and one bias (${\text{bias}}_{\text{m}}$) for the output neuron. Table 1 summarizes the correlation efficiency and significance between each category of the parameters and errors. The weights for the neurons in the hidden-layer ANN training using the BP and the GE-GA strongly correlated negatively with the fitting error; however, those in the other two ANNs did not. The bias for the output neuron has no significant correlation with the fitting errors in all four algorithms. All the output neuron weights in the ANN training with the GE-GA, BP, and MW strongly correlated with the errors (Fig. 1D); however, those in the FIB-GA ANN training did not. These results showed that in the ANN training with the FIB-GA, no parameter set correlated with the fitting error, a finding that implies there is no dominant parameter in ANNs trained via the use of an FIB-GA.

**Table 1:** Correlation between each category of parameters and average errors

ANN	BP ANN				MW ANN			
Parameter	$\mathcal{w}_{{\mathcal{MA}}}^{\mathcal{H}}$	$\mathcal{b}_{{\mathcal{MA}}}^{\mathcal{H}}$	$\mathcal{w}_{{\mathcal{MA}}}^{\mathcal{O}}$	$\mathcal{b}_{{}}^{\mathcal{O}}$	$\mathcal{w}_{{\mathcal{MA}}}^{\mathcal{H}}$	$\mathcal{b}_{{\mathcal{MA}}}^{\mathcal{H}}$	$\mathcal{w}_{{\mathcal{MA}}}^{\mathcal{O}}$	$\mathcal{b}_{{}}^{\mathcal{O}}$
r	−0.659	0.158	0.936	−0.152	0.051	0.018	0.857	0.064
p	0.002	0.507	0	0.521	0.831	0.940	0	0.788

ANN	GE-GA ANN				FIB-GA ANN			
Parameter	$\mathcal{w}_{{\mathcal{MA}}}^{\mathcal{H}}$	$\mathcal{b}_{{\mathcal{MA}}}^{\mathcal{H}}$	$\mathcal{w}_{{\mathcal{MA}}}^{\mathcal{O}}$	$\mathcal{b}_{{}}^{\mathcal{O}}$	$\mathcal{w}_{{\mathcal{MA}}}^{\mathcal{H}}$	$\mathcal{b}_{{\mathcal{MA}}}^{\mathcal{H}}$	$\mathcal{w}_{{\mathcal{MA}}}^{\mathcal{O}}$	$\mathcal{b}_{{}}^{\mathcal{O}}$
r	−0.789	−0.070	0.901	0.291	−0.260	0.524	0.339	−0.258
p	0	0.768	0	0.214	0.268	0.018	0.144	0.272

### The FT performance of ANNs in solving an XOR problem with a single fault

**Figure 2:**
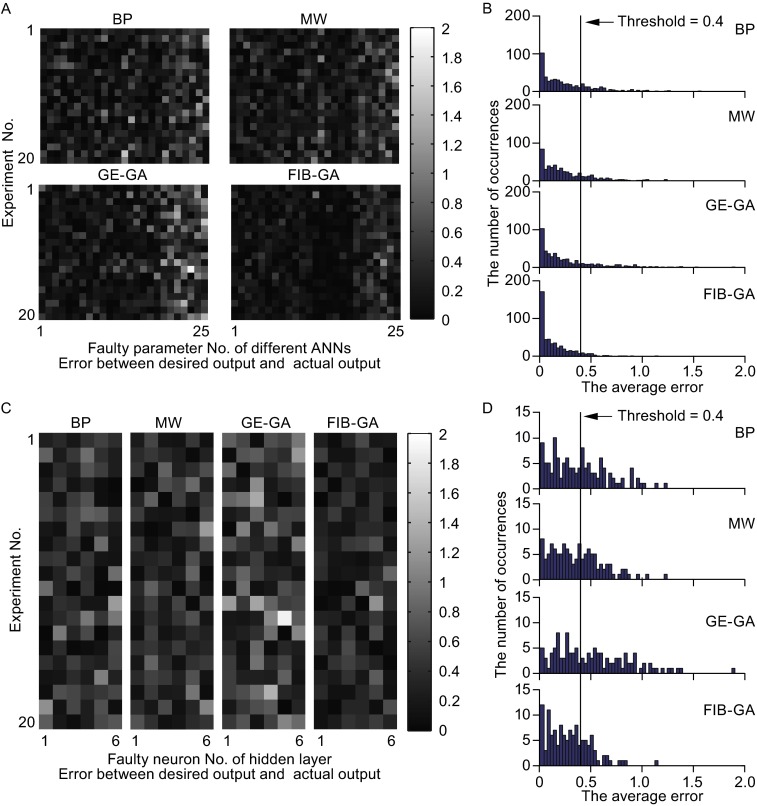
**The FT performance of ANNs in solving an XOR problem with a single faulty parameter or neuron in the hidden layer**. (A and B) The plot of errors versus the faulty parameter (A) and a histogram of error occurrence (B) in 20 independent experiments using ANNs trained via BP, MW, GE-GA, and FIB-GA methods. (C and D) The plot of errors versus the faulty neurons in the hidden layer (C) and a histogram of error occurrence (D) in 20 independent experiments using ANNs trained via BP, MW, GE-GA, and FIB-GA methods. In panels C and D, a fault output represents a fitting with the error equal to or exceeding a threshold of 0.4 (red lines)

Fault tolerance is the property that allows an ANN or BNN to operate properly in the event one or more components are lost. We began by comparing the errors among the ANNs generated by the BP, MW, GE-GA, and FIB-GA methods in which one randomly selected network parameter was changed to 0 (void). The plot of the errors versus the faulty parameters in 20 independent experiments clearly shows that the ANNs constructed using the FIB-GA contains the least number of errors (Fig. 2A). The averaged errors from 20 independent experiments are as follows: BP: 0.2623 ± 0.0614, MW: 0.2507 ± 0.0355, GE-GA: 0.2746 ± 0.0698, and FIB-GA: 0.1527 ± 0.0150 (statistical test in Table S2). Assuming the error equals or exceeds 0.4 as a fault output, the error rates show a similar trend (Fig. 2B): BP: 24.80 ± 9.68%, MW: 21.60 ± 7.94%, GE-GA: 23.80 ± 10.66%, and FIB-GA: 8.60 ± 5.24% (statistical test in Table S2). Next, the FT performances of the four ANNs were compared when one neuron (rather than one parameter) in the hidden layer completely lost its responsiveness. The performances of all four ANNs were reduced when compared with the fully functional ANNs, while the FIB-GA ANNs showed the fewest errors (Fig. 2C): BP: 0.3900 ± 0.1041, MW: 0.3645 ± 0.0567, GE-GA: 0.5167 ± 0.1413, and FIB-GA: 0.2936 ± 0.0410; (statistical test in Table S3) and the lowest error rates, with a 0.4 threshold (Fig. 2D): BP: 45.00 ± 19.57%, MW: 40.83 ± 13.76%, GE-GA: 54.17 ± 20.86%, and FIB-GA: 27.50 ± 13.55% (statistical test in Table S3).

As the output matrix is composed of four elements in the XOR problem, the errors of the individual elements were compared. The distributions of ${\text{Error}}_{-}{\text{c}}_{\text{i}}$ among the four ANNs were plotted while voiding one parameter or one neuron in the hidden layer (Fig. S2). Among the four algorithms, ANN training with the FIB-GA consistently showed the least number of errors (Fig. S2A): BP: 0.2623 ± 0.0614, MW: 0.2507 ± 0.0355, GE-GA: 0.2746 ± 0.0698, and FIB-GA: 0.1527 ± 0.0150 (statistical test in Table S4) and the lowest error rate (Fig. S2B): BP: 25.40 ± 7.94%, MW: 25.90 ± 5.39%, GE-GA: 25.75 ± 7.35%, and FIB-GA: 14.85 ± 3.30% (statistical test in Table S4). When one neuron in the hidden layer was voided randomly, the average error and the error rate with the 0.4 threshold showed a similar trend (Fig. S2C): average error: BP: 0.3900 ± 0.1041, MW: 0.3645 ± 0.0567, GE-GA: 0.5167 ± 0.1413, and FIB-GA: 0.2936 ± 0.0410 (statistical test in Table S5). Figure S2D shows the error rate with a 0.4 threshold: BP: 43.75 ± 17.34%, MW: 42.50 ± 12.72%, GE-GA: 48.13 ± 16.36%, and FIB-GA: 31.25 ± 6.55% (statistical test in Table S5). Together, these results clearly show that the FIB-GA ANN has superior FT when one parameter or one neuron in the network is lost.

### The FT performance of ANNs in solving an XOR problem with multiple faults

**Figure 3:**
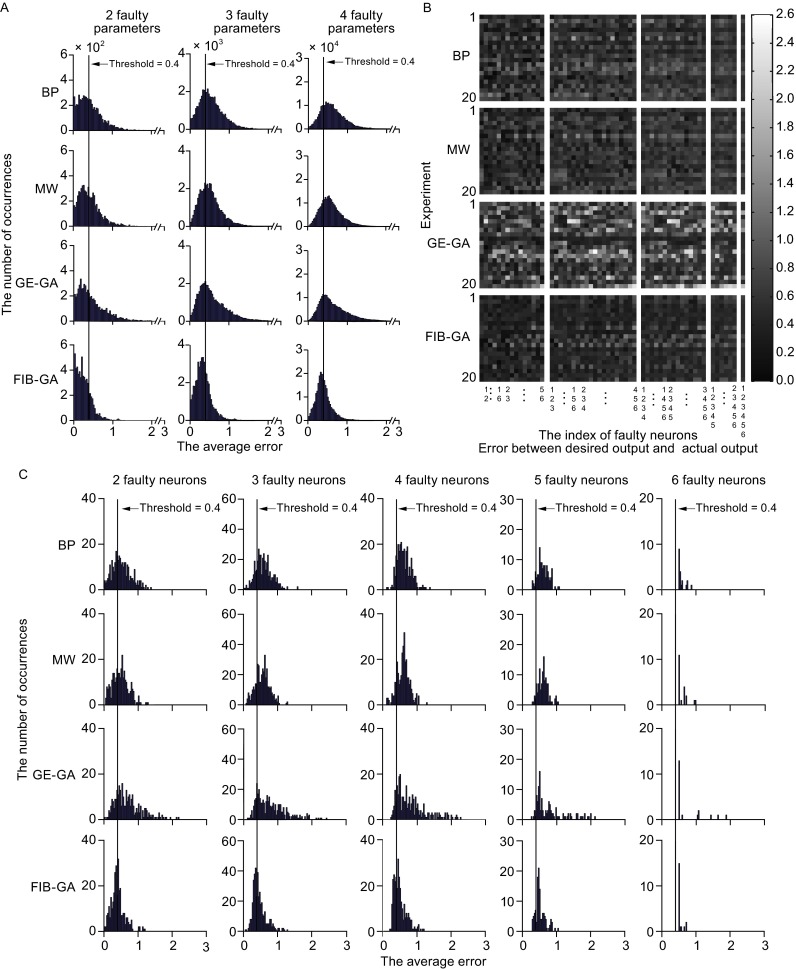
**The FT performance of ANNs in solving an XOR problem with multiple faulty parameters or neurons in the hidden layer**. (A) The histogram of error occurrence in 20 independent experiments using ANNs trained via BP, MW, GE-GA, and FIB-GA methods. (B and C) The plot of errors versus faulty neurons (B) and a histogram of error occurrence (C) in 20 independent experiments using ANNs trained via BP, MW, GE-GA, and FIB-GA methods

In both ANN and BNN, errors typically happen at multiple sites but are not restricted to one element. Thus, the performances of ANNs were compared to solve an XOR problem in which two to four parameters are disabled simultaneously. As each ANN has 25 parameters, there are 300 (${\text{C}}_{ 2 5}^{ 2}$), 2,300 (${\text{C}}_{ 2 5}^{ 3}$), and 12,650 (${\text{C}}_{ 2 5}^{ 4}$) combinations when two, three, and four parameters are all set to 0, respectively. Figure 3A illustrates the distribution of error; the summarized data clearly show that the FIB-GA-trained ANN still performed best under multiple-fault conditions (Table 2; statistical test in Tables S6–7). Next, we examined the errors occurring when two to six neurons in the hidden layer are voided. Under these circumstances, the ANN trained using the GE-GA showed the largest number of errors, while the ANN trained using the FIB-GA demonstrated the best performance (Fig. 3B). The error rates with a 0.4 threshold displayed the same order in the fitting performance (Fig. 3C). Not surprisingly, the performance of the ANNs trained using the FIB-GA was significantly better than the other three ANNs; however, the performance of the FIB-GA-trained ANNs weakened as the number of voided neurons increased (Table 3 and Fig. S3; statistical test in Tables S8–9). Since the performance of ANNs highly relies on the number of nodes in the hidden layer(Xu and Xu, 2013; Sasakawa et al., 2014). we next investigated whether the number of hidden neurons could affect the FT performance of the four ANNs in solving the XOR problem. In the ANNs with three or nine neurons in the hidden layer, the FIB-GA-trained ANN continued to demonstrate an FT performance that was superior to that of the BP, MW, and GE-GA ANNs (three neurons: Table 4; statistical test in Tables S10–11; nine neurons: Table 5; statistical test in Tables S12–13).

**Table 2:** Average errors and error rates of multiple faulty parameters with six hidden neurons

Error		BP	MW	GE-GA	FIB-GA
Average error	2	0.4295 ± 0.0925	0.4092 ± 0.0538	0.4534 ± 0.1092	0.2603 ± 0.0271
	3	0.5419 ± 0.1086	0.5158 ± 0.0649	0.5752 ± 0.1334	0.3392 ± 0.0373
	4	0.6199 ± 0.1167	0.5903 ± 0.0716	0.6609 ± 0.1490	0.3983 ± 0.0453
Error rate	2	47.03% ± 13.11%	45.03% ± 9.92%	45.45% ± 15.25%	20.05% ± 7.50%
	3	63.52% ± 12.68%	62.87% ± 9.52%	62.07% ± 15.54%	31.93% ± 9.06%
	4	74.61% ± 10.75%	74.31% ± 8.18%	73.43% ± 13.86%	43.29% ± 9.75%

**Table 3:** Average errors and error rates of multiple faulty neurons with six hidden neurons

Error		BP	MW	GE-GA	FIB-GA
Average error	2	0.5229 ± 0.1127	0.4891 ± 0.0794	0.7041 ± 0.1804	0.4077 ± 0.0657
	3	0.5960 ± 0.1231	0.5593 ± 0.0878	0.7891 ± 0.2036	0.4665 ± 0.0837
	4	0.6227 ± 0.1210	0.5974 ± 0.0969	0.8235 ± 0.2398	0.5009 ± 0.0909
	5	0.6115 ± 0.1174	0.6133 ± 0.1176	0.8096 ± 0.3082	0.5267 ± 0.0905
	6	0.5765 ± 0.1094	0.6037 ± 0.1489	0.7618 ± 0.4398	0.5382 ± 0.0740
Error rate	2	62.67% ± 12.31%	62.00% ± 13.87%	77.67% ± 18.00%	47.33% ± 15.58%
	3	80.00% ± 13.76%	78.00% ± 12.61%	81.75% ± 16.96%	55.25% ± 16.26%
	4	88.33% ± 14.00%	86.67% ± 11.03%	89.00% ± 13.90%	65.00% ± 14.49%
	5	90.83% ± 16.64%	94.17% ± 11.18%	95.00% ± 9.52%	81.67% ± 13.13%
	6	100.00% ± 0.00%	100.00% ± 0.00%	100.00% ± 0.00%	100.00% ± 0.00%

**Table 4:** Error rates of multiple faulty parameters and neurons with three hidden neurons

Error		BP	MW	GE-GA	FIB-GA
Parameters	1	33.85% ± 14.21%	41.15% ± 16.03%	38.85% ± 13.08%	8.46% ± 6.06%
	2	63.91% ± 12.94%	71.15% ± 13.27%	70.51% ± 13.50%	30.77% ± 7.51%
	3	82.52% ± 8.51%	87.13% ± 7.39%	86.00% ± 9.24%	56.59% ± 4.78%
Neurons	1	71.67% ± 16.31%	75.00% ± 18.34%	86.67% ± 19.94%	18.33% ± 17.01%
	2	95.00% ± 16.31%	95.00% ± 16.31%	88.33% ± 16.31%	46.67% ± 42.44%
	3	100.00% ± 0.00%	100.00% ± 0.00%	100.00% ± 0.00%	100.00% ± 0.00%

**Table 5:** Error rates of multiple faulty parameters and neurons with nine hidden neurons

Error		BP	MW	GE-GA	FIB-GA
Parameters	1	22.70% ± 9.62%	23.11% ± 8.01%	20.95% ± 11.29%	1.22% ± 1.38%
	2	46.67% ± 12.70%	47.70% ± 12.56%	38.20% ± 15.48%	4.44% ± 1.91%
	3	63.45% ± 13.11%	64.35% ± 12.40%	52.43% ± 16.25%	9.57% ± 2.85%
Neurons	1	41.67% ± 15.24%	45.00% ± 14.18%	48.89% ± 20.52%	1.67% ± 4.07%
	2	60.14% ± 16.05%	65.69% ± 14.20%	66.39% ± 17.40%	10.83% ± 6.62%
	3	71.43% ± 12.67%	76.31% ± 11.55%	75.42% ± 14.32%	20.89% ± 7.74%

Together, these results demonstrate that the FIB-GA ANN has superior FT in solving XOR problems when multiple parameters or neurons in the network are lost.

### The FT performance of ANNs in solving an overlapping classification problem

**Figure 4:**
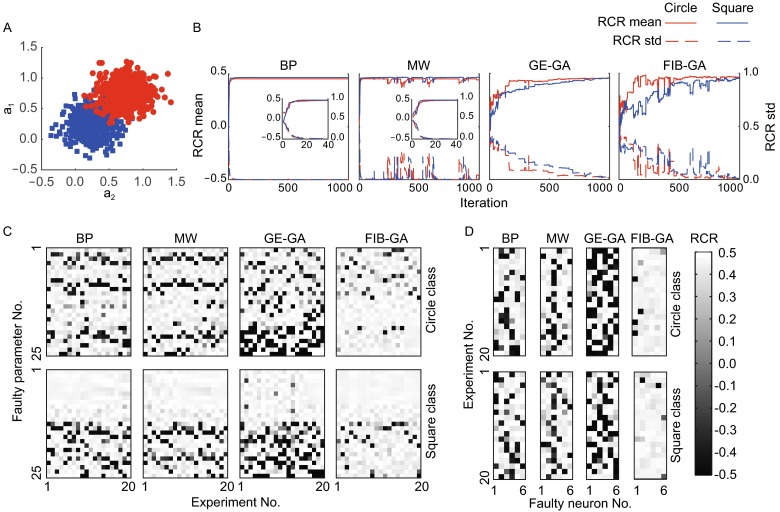
**The FT performance of different ANNs with six hidden-layer neurons in solving an overlapping classification problem**. (A) Two classes of Gaussian noise sources. (B) The plot of the relative correct rate versus the number of iterations. The inset shows the changes of the error within the initial 40 iterations. (C and D) The plot of errors versus the faulty parameters (C) and the plot of errors versus the faulty neurons in the hidden layer (D) in 20 independent experiments when using ANNs trained via BP, MW, GE-GA, and FIB-GA methods

**Figure 5:**
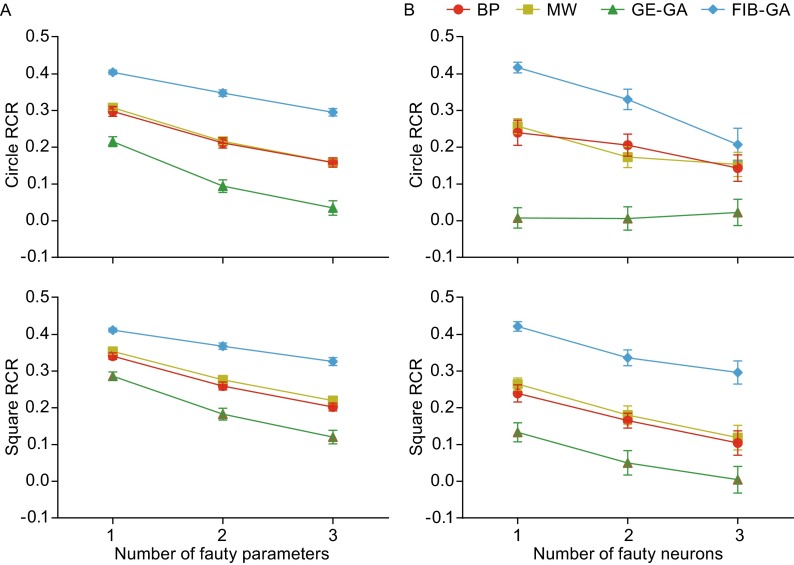
**Relative correct rates of different ANNs with six hidden-layer neurons in solving an overlapping classification problem**. (A) The plot of circle RCR (top) and square RCR (bottom) versus the number of faulty parameters. (B) The plot of circle RCR (top) and square RCR (bottom) versus the number of faulty neurons

Next, we examined the FT performance of the four ANNs in solving an overlapping classification problem (Fig. 4A) that is more complicated than an XOR problem. Solving overlapping classification problems using ANNs has been investigated extensively in the pattern recognition and machine-learning areas (Lovell and Bradley, 1996; Tang et al., 2010; Xiong et al., 2010). We adopted an ANN with the same structures used in these previous studies (see Fig. 1A). The terminating condition was set at 1,000 iterations, since none of the four ANNs could satisfy the condition that the fitting error must be equal to or less than 0.001 within 1,000 iterations, partly due to the complexity of the problem. Figure 4B illustrates the changes in the correct rates versus the number of iterations. The correct rates of BP and MW ANNs increased significantly faster compared with those of GE-GA and FIB-GA (fitting function: ${\text{f}}({\text{x}}) = {\text{a}} \cdot {\text{e}}^{{ - \tau {\text{x}}}}$, square class RCR: BP: 0.4662 ± 0.0006, $\tau$ = 0.2717, $R^{2}$ = 0.9880, MW: 0.4672 ± 0.0010, $\tau$ = 0.2529, $R^{2}$ = 0.8855, GE-GA: 0.4605 ± 0.0166, $\tau$ = 0.0040, $R^{2}$ = 0.9068, FIB-GA: 0.4578 ± 0.0083, $\tau$ = 0.0030, $R^{2}$ = 0.8502, and circle class RCR: BP: 0.4542 ± 0.0006, $\tau$ = 0.2210, $R^{2}$ = 0.9230, MW: 0.4541 ± 0.0115, $\tau$ = 0.2581, $R^{2}$ = 0.8996, GE-GA: 0.4547 ± 0.0115, $\tau$ = 0.0084, $R^{2}$ = 0.9014, FIB-GA: 0.4605 ± 0.0074, $\tau$ = 0.0111, $R^{2}$ = 0.7846 (statistical test in Table S14). The number of iterations that occurred when RCR reached 0.45 are shown for each method as follows: BP 23.2 ± 14.3, MW 15.8 ± 8.3, GE-GA 613.7 ± 341.8, and FIB-GA 583.1 ± 287.7. We then examined the FT performance of these four ANNs in solving an overlapping classification problem and found that the FIB-GA ANN showed significantly fewer errors when one parameter or one neuron was voided. When any of the parameters were voided one at a time in 20 independent experiments, the square class RCRs for FIB-GA ANN was the highest compared with the other three ANNs, while the other three ANNs were not significantly different (square class RCRs: BP: 0.3407 ± 0.0397, MW: 0.3536 ± 0.0317, GE-GA: 0.2863 ± 0.0501, and FIB-GA: 0.4116 ± 0.0247; circle class RCRs: BP: 0.2978 ± 0.0600, MW: 0.3075 ± 0.0301, GE-GA: 0.2151 ± 0.0601, and FIB-GA: 0.4045 ± 0.0258 (statistical test in Table S15) (Fig. 4C). When one neuron in the hidden layer was voided randomly, FIB-GA ANN still significantly outperformed the other three ANNs (square class RCRs: BP: 0.2392 ± 0.1049, MW: 0.2646 ± 0.0743, GE-GA: 0.1334 ± 0.1160, and FIB-GA: 0.4224 ± 0.0586; circle class RCRs: BP: 0.2396 ± 0.1523, MW: 0.2574 ± 0.0911, GE-GA: 0.0073 ± 0.1242, and FIB-GA: 0.4164 ± 0.0640 (statistical test in Table S16) (Fig. 4D). We next randomly voided two to three parameters or neurons in these ANNs and compared their FT performance. As illustrated in Figure 5, voiding two to three parameters or neurons reduced the performance of all four ANNs. The FIB-GA showed the fewest errors and lowest error rates under almost all the fault conditions tested (Tables S17–18). Thus, our data clearly demonstrate that, compared to the ANNs trained using the BP, MW, and GE-GA methods, the FT ability of the ANN trained using the FIB-GA, at a relatively low training speed, is superior.

## Discussion

This study compared the FT performances of ANNs constructed with four different algorithms: BP, MW, GE-GA, and FIB-GA. The FIB-GA was constructed via the use of FIB learning algorithms, which has been proven to be a common and efficient method of training fault-tolerant neural networks that includes the addition of noise to the input, weights, or nodes (Leung and Sum, 2008; Ho et al., 2010). Our results clearly show that the FIB learning algorithm is an efficient method for improving the FT performance of ANNs. The data of this study show that FIB-GA results in a significant improvement in errors between the actual and desired inputs when one or multiple neurons are voided. It is worth noting that, when solving an XOR problem or an overlapping classification problem with the continuous and differentiable basis function, the GE-GA does not offer an advantage over the BP and MW algorithms. This might suggest that FT is not an intrinsic property of GA ANNs. In contrast, compared to the two GAs, the BP and MW algorithms showed much faster training speeds, which implies that efficiency competes with fault tolerance in ANNs. An option for increasing the FT performances of ANNs is to avoid weights or neurons with significant effects on errors. Our analysis showed that the weights between the hidden layer and the output layer in the ANNs trained with the GE-GA, BP, or MW, but not those trained with the FIB-GA, are correlated with the output errors, a finding which clearly supports the notion that robustness is greater in a distributed ANN. This is also consistent with previous attempts to improve the partial FT of ANNs by distributing the absolute value of weights uniformly (Cavalieri and Mirabella, 1999a, b). In addition, Macia and Sole reported that degeneracy, rather than redundancy, is necessary for reliable designs of NAND (NOT AND, a binary operation) gate-forming systems(Macia and Sole, 2009). Considering the fact that the BNNs are also distributed systems yielding a high FT performance, distributed storage and processing seem to be key properties in both ANNs and BNNs. Together, our results propose that a fault/noise injection-based genetic algorithm would serve as an efficient approach for improving the FT in ANNs.

## Methods

### The architecture of the ANN

In this study, a three-layer ANN was constructed: an input layer of two neurons, a hidden layer comprised of different numbers of neurons, and an output layer of one neuron. Figure 1A shows the architecture. Each neuron receives multiple weighted inputs and sends one weighted output to the connected neurons. Simply stated, neurons are interconnected through a unidirectional manner (input → hidden → output direction), and there is no connection within a layer (Fig. 1A). The input of the neuron *j* in the hidden layer is given by

$${\text{x}}_{\text{j}} = \mathop \sum \limits_{{{\text{i}} = 1}}^{\text{n}} {\left( {{\text{Input}}_{\text{ij}} \times {\text{weight}}_{\text{ij}} } \right) + {\text{b}}_{\text{j}} }$$

where ${\text{Input}}_{\text{ij}}$, ${\text{weight}}_{\text{ij}}$, and ${\text{b}}_{\text{j}}$ denote the input, weight, and input bias of the postsynaptic neuron *j*, which is connected to the presynaptic neuron *i*, and n denotes the number of the presynaptic neurons connecting to the neuron *j*.

The output of the neuron *j* is given by the following tansig function:

$${\text{y}}_{\text{j}} = {\text{f}}\left( {{\text{x}}_{\text{j}} } \right) = \frac{{ 1- {\text{e}}^{{ - 2 {\text{x}}_{\text{j}} }} }}{{ 1+ {\text{e}}^{{ - 2 {\text{x}}_{\text{j}} }} }}$$

### Calculation of the ANN to solve the XOR problem

A classic XOR problem was selected for use in training and examining the performance of the ANN. According to the architecture of the ANN used in this study (Fig. 1A), the training data, ${\text{a}}_{ 1}$ and ${\text{a}}_{ 2}$, are defined as follows:

$${\text{a}}_{ 1} = \left[ {\begin{array}{*{20}c} 1\quad& 0\quad& 1\quad& 0\\ \end{array} } \right]$$

$${\text{a}}_{ 2} = \left[ {\begin{array}{*{20}c} 1\quad& 1\quad& 0\quad& 0\\ \end{array} } \right]$$

Thus, the actual output of the XOR problem with these two inputs is given by

$${\text{y}} = \left[ {\begin{array}{*{20}c} {{\text{y}}_{ 1} }\quad & {{\text{y}}_{ 2} }\quad & {{\text{y}}_{ 3} }\quad & {{\text{y}}_{ 4} } \\ \end{array} } \right] = {\text{a}}_{ 1} \oplus {\text{a}}_{ 2} = \left( {\neg {\text{a}}_{ 1} \wedge {\text{a}}_{ 2} } \right) \vee \left( {{\text{a}}_{ 1} \wedge \neg {\text{a}}_{ 2} } \right) = \left[ {\begin{array}{*{20}c} 0\quad& 1\quad& 1\quad& 0\\ \end{array} } \right].$$

For one solution set, the output of the ANN is given by

$${\text{c}} = \left[ {\begin{array}{*{20}c} {{\text{c}}_{ 1} }\, & {{\text{c}}_{ 2} }\, & {{\text{c}}_{ 3} }\, & {{\text{c}}_{ 4} }\, \\ \end{array} } \right]$$

where ${\text{c}}_{\text{p}} = \sum_{{{\text{j}} = 1}}^{ 6} {\left( {{\text{f}}\left( {{\text{x}}_{\text{j}} } \right) \times {\text{weight}}_{\text{jm}} } \right) + {\text{b}}_{\text{m}} }$ and ${\text{x}}_{\text{j}}$ denote the input to the neuron *j* in the hidden layer from the two presynaptic neurons (${\text{a}}_{ 1}$ and ${\text{a}}_{ 2}$), which is given by ${\text{x}}_{\text{j}} = \sum_{{{\text{i}} = 1}}^{ 2} {\left( {{\text{a}}_{\text{i}} \left( {\text{p}} \right) \times {\text{weight}}_{\text{ij}} } \right) + {\text{b}}_{\text{j}} }$.and ${\text{Weight}}_{\text{jm}}$ and ${\text{b}}_{\text{m}}$ denote the weight and bias of the neuron $m$ in the output layer, respectively.

Thus, the error for one solution set is given by

$${\text{Error}} = \frac{1}{4}\mathop \sum \limits_{{{\text{p}} = 1}}^{ 4} {\left| {{\text{c}}_{\text{p}} } - {{\text{y}}_{\text{p}} }\right|}$$

### Training of the ANN using a GE-GA

A GE-GA was adopted for training the ANN. The basic idea of a GA is to mimic the process of natural selection and to find the best solution to a problem after several generations. In this study, the upper limit of iteration was set at 1,000, and 20 individuals (i.e., sets of solutions) were used in each generation (i.e., training cycle). The best individual is defined as the one set of solutions having minimum errors. In the first generation, each individual was assigned randomly. In the subsequent generations, the 20 individuals consisted of three parts: two elite individuals (${\text{N}}_{\text{elite}}$), which are the two individuals carried forward from the previous generation and having the fewest errors, 14 crossover individuals (${\text{N}}_{\text{crossover}}$), which are generated by combining two selected parents, and four mutation individuals (${\text{N}}_{\text{mutation}}$). The selection criteria for parents is based on the scaled position (${\text{Scaled}}_{\text{i}}$) of each individual within its generation. ${\text{Rank}}_{\text{i}}$ is redefined as the position of ${\text{Individual}}_{\text{i}}$ when sorting all the individuals in one generation by ${\text{Error}}$ in ascending order. Thus, for individual i, the probability to be selected as a parent is calculated as follows:

$${\text{P}}_{\text{i}} = {{{\text{Scaled}}_{\text{i}} } \mathord{\left/ {\vphantom {{{\text{Scaled}}_{\text{i}} } {\sum_{{{\text{j}} = 1}}^{ 2 0} {\frac{ 1}{{\sqrt {\text{j}} }}} }}} \right.} {\mathop \sum \limits_{{{\text{j}} = 1}}^{ 2 0} {\frac{ 1}{{\sqrt {\text{j}} }}} }} = {\text{Scaled}}_{\rm i}/7.5953$$

where $${\text{Scaled}}_{\text{i}} = { 1{\left/ {\vphantom { 1{\sqrt {{\text{Rank}}_{\text{i}} } }}} \right.} {\sqrt {{\text{Rank}}_{\text{i}} } }}$$, ($${\text{Rank}}_{\text{i}} \ne {\text{Rank}}_{\text{j}}\, {\text{if}}\;{\text{i}} \ne {\text{j}}$$).

A line segment was then drawn that consisted of lines whose lengths were proportional to the ${\text{P}}_{\text{i}}$ of each individual. A step size was given by $1/{\text{N}}_{{\rm parent}}$, where ${\text{N}}_{\text{parent}} = 2\cdot {\text{N}}_{\text{crossover}} + {\text{N}}_{\text{mutation}} = 2\times 1 4+ 4= 3 2$ and an initial position is denoted as ${\text{Initial}}_{\text{position}}$, where $0< {\text{Initial}}_{\text{position}} < 1/{\text{N}}_{{\rm parent}}$. A cursor is then placed at ${\text{Initial}}_{\text{position}}$ and is moved along in steps of $1/{\text{N}}_{{\rm parent}}$. For each step, the position on which the cursor lands is selected as a parent. Thus, this algorithm generates 32 parents in one generation (Fig. S4). The crossover process generates a child by crossing two parents (${\text{Parent}}_{ 1} = \left[ {{\text{parP1}}_{ 1} ,\cdots \cdots , {\text{parP1}}_{ 2 5} } \right]$ and ${\text{Parent}}_{ 2} = \left[ {{\text{parP2}}_{ 1} ,\cdots \cdots , {\text{parP2}}_{ 2 5} } \right]$) with a randomly generated binary vector ${\text{Coef}} = \left[ {{\text{Coe}}_{ 1} ,\cdots \cdots , {\text{Coe}}_{ 2 5} } \right]$, where ${\text{Coe}}_{\text{i}}$ is assigned to 0 or 1 based on rounding a value that is randomly selected in the open interval (0,1). The parameter vector of the ${\text{Child}} = \left[ {{\text{parC}}_{ 1} ,\cdots \cdots , {\text{parC}}_{ 2 5} } \right]$ generated by the crossover is given by

$${\text{parC}}_{\text{i}} = {\text{parP1}}_{\text{i}} \times {\text{Coe}}_{\text{i}} + {\text{parP2}}_{\text{i}} \times \left| {{\text{Coe}}_{\text{i}} - 1} \right|.$$

The mutation process generates a child from one parent (${\text{Parent}}_{ 1} = \left[ {{\text{parP1}}_{ 1} ,\cdots \cdots , {\text{parP1}}_{ 2 5} } \right]$) with a vector ${\text{Coef}} = \left[ {{\text{Coe}}_{ 1} ,\cdots \cdots , {\text{Coe}}_{ 2 5} } \right]$, where $Coe_{i}$ follows a Gaussian distribution centered at 0 (Fig. S4C). The standard deviation of the Gaussian distribution in the first generation is 1, and it shrinks to 0 linearly when reaching the last generation. The parameter vector of the ${\text{Child}} = \left[ {{\text{parC}}_{ 1} ,\cdots \cdots , {\text{parC}}_{ 2 5} } \right]$ generated by the mutation is given by

$${\text{parC}}_{\text{i}} = {\text{parP1}}_{\text{i}} + {\text{Coe}}_{\text{i}} .$$

The goal of training the ANN with a GE-GA is to search for the individual with the minimal ${\text{Error}}_{{{\text{GE}} - {\text{GA}}}}$which is given by

1 $${\text{Error}}_{{\text{GE}} - {\text{GA}}} = \frac{1}{4} \mathop \sum \limits_{{\text{p}} = 1}^{4}|{\text{c}}_{\rm p}-{\text{y}}_{\rm p}|$$

### Training of ANN using with a FIB-GA

In addition to the GE-GA, a FIB-GA was another approach used to train the ANN. In the FIB-GA, faults on the ANN parameters were considered during the training process. Thus, the error for one set of solutions is given by

2 $${\text{Error}}_{{{\text{FIB}} - {\text{GA}}}} = \frac{ 1}{ 2 5} \times \mathop \sum \limits_{{{\text{p}} = 1}}^{ 2 5} {{\text{Error}}_{\text{i}} }$$

where ${\text{Error}}_{\text{i}}$ is the error when the ${\text{i}}^{\text{th}}$ of the 25 parameters is forced to 0, assuming the corresponding parameter becomes faulty.

### Overlapping classification problem

Two classes of Gaussian noise sources were considered (shown in Fig. 4A). The first class is shown as a blue square, with a mean at ($a_{1} ,\;a_{2}$) coordinates of (0.25, 0.25). The second class is shown as a red circle, with a mean at ($a_{1} ,\;a_{2}$) coordinates of (0.75, 0.75). Both classes have a standard deviation of 0.2. The coordinates ($a_{1} ,\;a_{2}$) were used as input in the ANN training, and the output is 0.5 and −0.5 for the circle class and the square class, respectively. Each class had 500 scatters in total, and all the data were shuffled before the training was initiated. An actual output value larger than 0 for a point in the circle class and an actual output value less than 0 for a point in the square class were regarded correct.

For each class with N ($0 \le {\text{N}} \le 500$) points classified correctly, the relative correct rate (RCR) is defined as follows:

$${\text{RCR}} = \frac{\text{N}}{500}\, - 0.5.$$

## Electronic supplementary material

Below is the link to the electronic supplementary material.

Supplementary material 1 (PDF 609 kb)
