# Supplementary material for: The superior fault tolerance of artificial neural network training with a fault/noise injection-based genetic algorithm
Source: Protein Cell. 2016 Aug 9;7(10):735–48. doi: 10.1007/s13238-016-0302-5 (PMC5055486; doi:10.1007/s13238-016-0302-5)
Supplement: Supplementary file 1 — Supplementary material 1 (PDF 609 kb) [file 13238_2016_302_MOESM1_ESM.pdf]

Supplementary materials

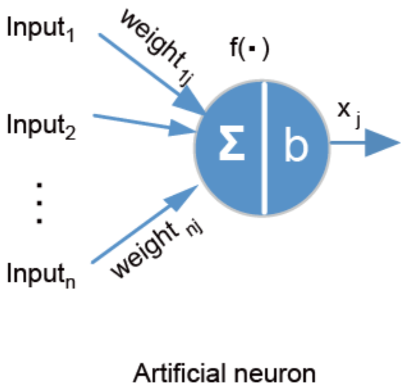

Supplementary Fig. S1. The calculation model of an artificial neuron.

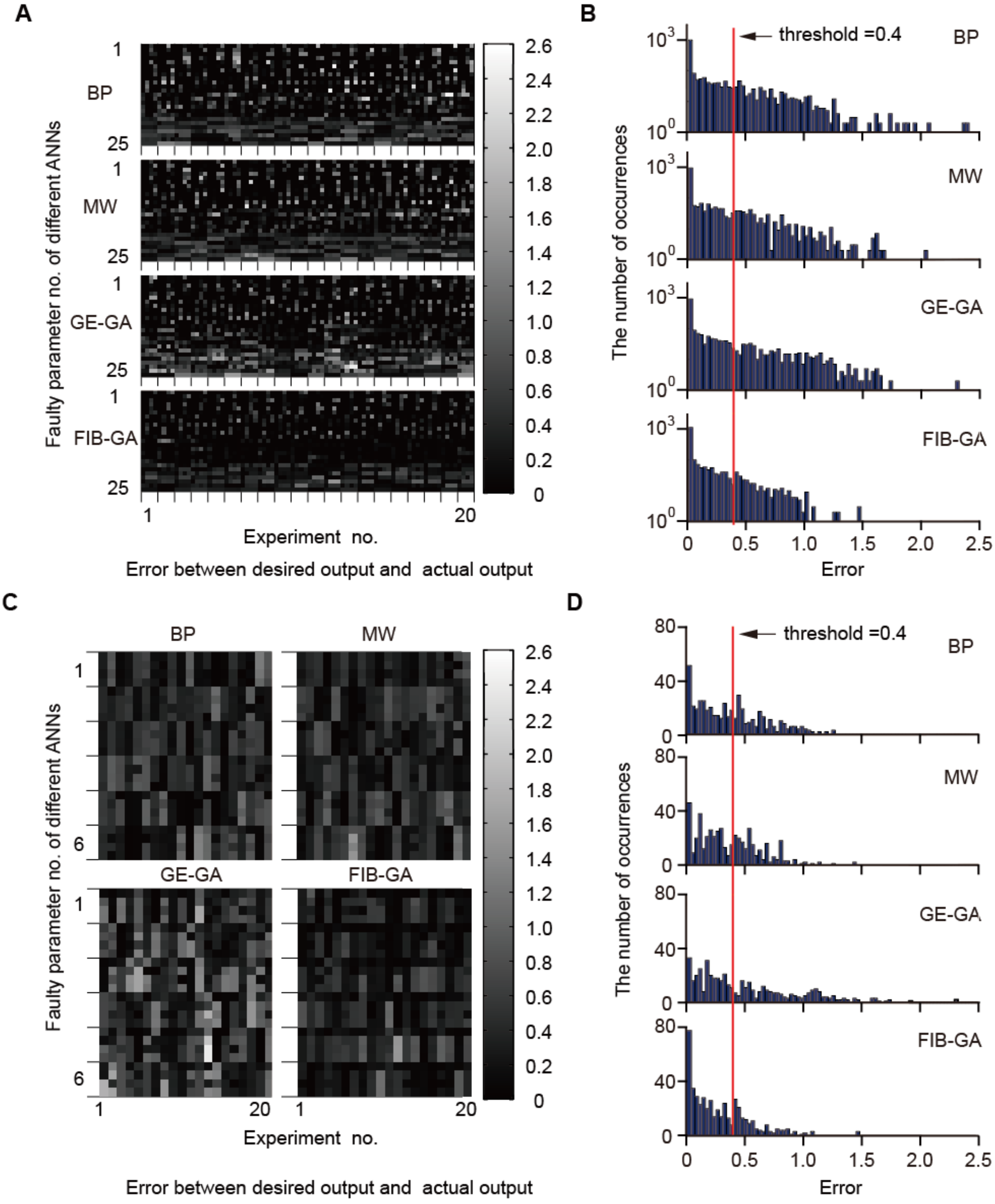

Supplementary Fig. S2. The FT performance of ANNs in solving an XOR problem with a single fault parameter or neuron in the hidden layer. For each experiment, there are four elements in the output with a faulty parameter or a faulty neuron. (A–B) The plot of error versus the faulty parameter (A) and histogram of error occurrence (B) in 20 independent experiments using BP, MW, GE-GA, and FIB-GA

ANNs. (C–D) The plot of error versus the faulty neuron in the hidden layer (C) and histogram of error occurrence (D) in 20 independent experiments using BP, MW, GE-GA, and FIB-GA ANNs. In panel B and D, a fault output represented a fitting with the error equals or exceeds a threshold of 0.4 (red lines).

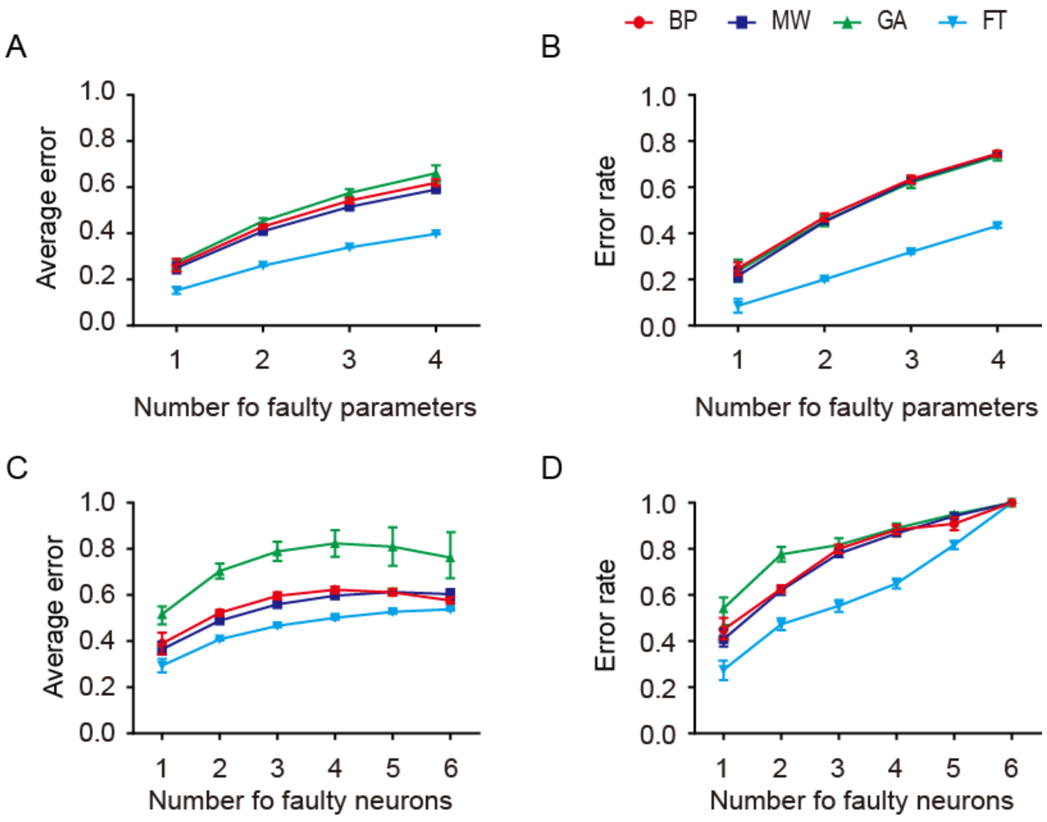

Supplementary Fig. S3. Average error and correct rate for ANN with six hidden neurons in solving XOR problem. Average error (A) and error rate (B) plot against with the number of faulty parameters. Average error (C) and error rate (D) plot against with the number of faulty neurons.

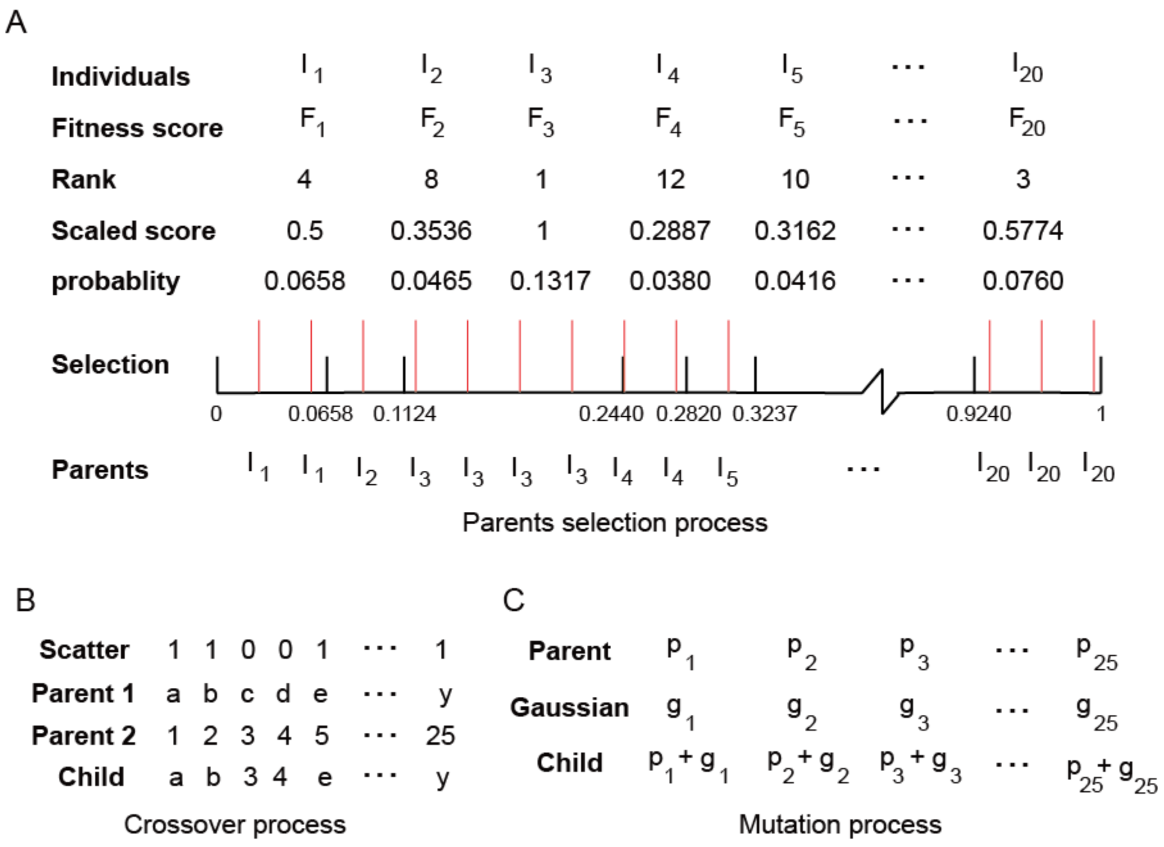

Supplementary Fig. S4. Several processes in the GA. (A) Parent selection processes. (B) Crossover process. (C) Mutation process.

Supplementary Table S1 Statistic test of ANN training results

Method	BP	MW	GE-GA	FIB-GA
BP	—	ns	(p = 0.005)**	(p = 0.0002)***
MW	—	—	(p = 0.005)**	(p = 0.0002)***
GE-GA	—	—	—	(p = 0.0068)**
FIB-GA	—	—	—	—

Supplementary Table S2 Statistic test of average error and error rate with one faulty parameter

error	method	BP	MW	GE-GA	FIB-GA
Average error	BP	—	(p=0.4690)ns	(p=0.5575)ns	(p<0.0001)***
	MW	—	—	(p=0.1803)ns	(p<0.0001)***
	GE-GA	—	—	—	(p<0.0001)***
	FIB-GA	—	—	—	—
Error rate	BP	—	(p=0.2602)ns	(p=0.7578)ns	(p<0.0001)***
	MW	—	—	(p=0.4637)ns	(p<0.0001)***
	GE-GA	—	—	—	(p<0.0001)***
	FIB-GA	—	—	—	—

Supplementary Table S3 Statistic test of average error and error rate with one faulty neuron

error	method	BP	MW	GE-GA	FIB-GA
Average error	BP	—	(p=0.3421)ns	(p=0.0026)**	(p=0.0004)***
	MW	—	—	(p<0.0001)***	(p<0.0001)***
	GE-GA	—	—	—	(p<0.0001)***
	FIB-GA	—	—	—	—
Error rate	BP	—	(p=0.4405)ns	(p=0.1598)ns	(p=0.0022)**
	MW	—	—	(p=0.0221)*	(p=0.0038)**
	GE-GA	—	—	—	(p<0.0001)***
	FIB-GA	—	—	—	—

Supplementary Table S4 Statistic test of average error and error rate for each element with one faulty parameter

error	method	BP	MW	GE-GA	FIB-GA
Average error	BP	—	(p=0.4690)ns	(p=0.5575)ns	(p<0.0001)***
	MW	—	—	(p=0.1803)ns	(p<0.0001)***
	GE-GA	—	—	—	(p<0.0001)***
	FIB-GA	—	—	—	—
Error rate	BP	—	(p=0.8170)ns	(p=0.8857)ns	(p<0.0001)***
	MW	—	—	(p=0.9417)ns	(p<0.0001)***
	GE-GA	—	—	—	(p<0.0001)***
	FIB-GA	—	—	—	—

Supplementary Table S5 Statistic test of average error and error rate each element with one faulty neuron

error	method	BP	MW	GE-GA	FIB-GA
Average error	BP	—	(p=0.3421)ns	(p=0.0026)**	(p=0.0004)***
	MW	—	—	(p<0.0001)***	(p<0.0001)***
	GE-GA	—	—	—	(p<0.0001)***
	FIB-GA	—	—	—	—
Error rate	BP	—	(p=0.7963)ns	(p=0.4164)ns	(p=0.0046)**
	MW	—	—	(p=0.2319)ns	(p=0.0012)**
	GE-GA	—	—	—	(p=0.0001)***
	FIB-GA	—	—	—	—

Supplementary Table S6 Statistic test of average error of multiple faulty parameters

Number	method	BP	MW	GE-GA	FIB-GA
2	BP	—	(p=0.4015)ns	(p=0.4597)ns	(p<0.0001)***
	MW	—	—	(p=0.1127)ns	(p<0.0001)***
	GE-GA	—	—	—	(p<0.0001)***
	FIB-GA	—	—	—	—
3	BP	—	(p=0.3620)ns	(p=0.3921)ns	(p<0.0001)***
	MW	—	—	(p=0.0813)ns	(p<0.0001)***
	GE-GA	—	—	—	(p<0.0001)***
	FIB-GA	—	—	—	—
4	BP	—	(p=0.3397)ns	(p=0.3388)ns	(p<0.0001)***
	MW	—	—	(p=0.0637)ns	(p<0.0001)***
	GE-GA	—	—	—	(p<0.0001)***
	FIB-GA	—	—	—	—

Supplementary Table S7 Statistic test of error rate of multiple faulty parameters

Number	method	BP	MW	GE-GA	FIB-GA
2	BP	—	(p=0.5896)ns	(p=0.7273)ns	(p<0.0001)***
	MW	—	—	(p=0.9183)ns	(p<0.0001)***
	GE-GA	—	—	—	(p<0.0001)***
	FIB-GA	—	—	—	—
3	BP	—	(p=0.8555)ns	(p=0.7482)ns	(p<0.0001)***
	MW	—	—	(p=0.8454)ns	(p<0.0001)***
	GE-GA	—	—	—	(p<0.0001)***
	FIB-GA	—	—	—	—
4	BP	—	(p=0.9214)ns	(p=0.9636)ns	(p<0.0001)***
	MW	—	—	(p=0.9736)ns	(p<0.0001)***
	GE-GA	—	—	—	(p<0.0001)***
	FIB-GA	—	—	—	—

Supplementary Table S8 Statistic test of average error of multiple faulty neurons

Number	method	BP	MW	GE-GA	FIB-GA
2	BP	—	(p=0.2798)ns	(p=0.0005)***	(p=0.0003)***
	MW	—	—	(p<0.0001)***	(p=0.0011)**
	GE-GA	—	—	—	(p<0.0001)***
	FIB-GA	—	—	—	—
3	BP	—	(p=0.2845)ns	(p=0.0008)***	(p=0.0004)***
	MW	—	—	(p<0.0001)***	(p=0.0015)**
	GE-GA	—	—	—	(p<0.0001)***
	FIB-GA	—	—	—	—
4	BP	—	(p=0.4699)ns	(p=0.0019)**	(p=0.0009)***
	MW	—	—	(p=0.004)***	(p=0.0024)**
	GE-GA	—	—	—	(p<0.0001)***
	FIB-GA	—	—	—	—
5	BP	—	(p=0.9616)ns	(p=0.0107)*	(p=0.0146)*
	MW	—	—	(p=0.0113)*	(p=0.0129)*
	GE-GA	—	—	—	(p=0.0003)***
	FIB-GA	—	—	—	—
6	BP	—	(p=0.5143)ns	(p=0.0753)ns	(p=0.2025)ns
	MW	—	—	(p=0.1361)ns	(p=0.0862)ns
	GE-GA	—	—	—	(p=0.0309)*
	FIB-GA	—	—	—	—

Supplementary Table S9 Statistic test of error rate of multiple faulty neurons

Number	method	BP	MW	GE-GA	FIB-GA
2	BP	—	(p=0.8725)ns	(p=0.0039)**	(p=0.0014)**
	MW	—	—	(p=0.0038)**	(p=0.0032)**
	GE-GA	—	—	—	(p<0.0001)***
	FIB-GA	—	—	—	—
3	BP	—	(p=0.6345)ns	(p=0.7221)ns	(p<0.0001)***
	MW	—	—	(p=0.4324)ns	(p<0.0001)***
	GE-GA	—	—	—	(p<0.0001)***
	FIB-GA	—	—	—	—
4	BP	—	(p=0.6794)ns	(p=0.8801)ns	(p<0.0001)***
	MW	—	—	(p=0.5605)ns	(p<0.0001)***
	GE-GA	—	—	—	(p<0.0001)***
	FIB-GA	—	—	—	—
5	BP	—	(p=0.4608)ns	(p=0.3368)ns	(p=0.0608)ns
	MW	—	—	(p=0.8018)ns	(p=0.0025)**
	GE-GA	—	—	—	(p=0.0007)***
	FIB-GA	—	—	—	—
6	BP	—	—	—	—
	MW	—	—	—	—
	GE-GA	—	—	—	—
	FIB-GA	—	—	—	—

Supplementary Table S10 Statistic test of error rate of multiple faulty parameters with 3 hidden neurons

Number	method	BP	MW	GE-GA	FIB-GA
1	BP	—	(p=0.1358)ns	(p=0.2542)ns	(p<0.0001)***
	MW	—	—	(p=0.6219)ns	(p<0.0001)***
	GE-GA	—	—	—	(p<0.0001)***
	FIB-GA	—	—	—	—
2	BP	—	(p=0.0887)ns	(p=0.1228)ns	(p<0.0001)***
	MW	—	—	(p=0.8806)ns	(p<0.0001)***
	GE-GA	—	—	—	(p<0.0001)***
	FIB-GA	—	—	—	—
3	BP	—	(p=0.0752)ns	(p=0.2230)ns	(p<0.0001)***
	MW	—	—	(p=0.6717)ns	(p<0.0001)***
	GE-GA	—	—	—	(p<0.0001)***
	FIB-GA	—	—	—	—

Supplementary Table S11 Statistic test of error rate of multiple faulty neurons with 3 hidden neurons

Number	method	BP	MW	GE-GA	FIB-GA
1	BP	—	(p=0.5476)ns	(p=0.0131)*	(p<0.0001)***
	MW	—	—	(p=0.0616)ns	(p<0.0001)***
	GE-GA	—	—	—	(p<0.0001)***
	FIB-GA	—	—	—	—
2	BP	—	(p>0.9999)ns	(p=0.2037)ns	(p<0.0001)***
	MW	—	—	(p=0.2037)ns	(p<0.0001)***
	GE-GA	—	—	—	(p=0.0002)***
	FIB-GA	—	—	—	—
3	BP	—	—	—	—
	MW	—	—	—	—
	GE-GA	—	—	—	—
	FIB-GA	—	—	—	—

Supplementary Table S12 Statistic test of error rate of multiple faulty parameters with 9 hidden neurons

Number	method	BP	MW	GE-GA	FIB-GA
1	BP	—	(p=0.8843)ns	(p=0.6008)ns	(p<0.0001)***
	MW	—	—	(p=0.4895)ns	(p<0.0001)***
	GE-GA	—	—	—	(p<0.0001)***
	FIB-GA	—	—	—	—
2	BP	—	(p=0.7979)ns	(p=0.0662)ns	(p<0.0001)***
	MW	—	—	(p=0.0396)*	(p<0.0001)***
	GE-GA	—	—	—	(p<0.0001)***
	FIB-GA	—	—	—	—
3	BP	—	(p=0.8247)ns	(p=0.0235)*	(p<0.0001)***
	MW	—	—	(p=0.0129)*	(p<0.0001)***
	GE-GA	—	—	—	(p<0.0001)***
	FIB-GA	—	—	—	—

Supplementary Table S13 Statistic test of error rate of multiple faulty neurons with 9 hidden neurons

Number	method	BP	MW	GE-GA	FIB-GA
1	BP	—	(p=0.4787)ns	(p=0.2142)ns	(p<0.0001)***
	MW	—	—	(p=0.4898)ns	(p<0.0001)***
	GE-GA	—	—	—	(p<0.0001)***
	FIB-GA	—	—	—	—
2	BP	—	(p=0.2540)ns	(p=0.2450)ns	(p<0.0001)***
	MW	—	—	(p=0.8899)ns	(p<0.0001)***
	GE-GA	—	—	—	(p<0.0001)***
	FIB-GA	—	—	—	—
3	BP	—	(p=0.2180)ns	(p=0.3566)ns	(p<0.0001)***
	MW	—	—	(p=0.8299)ns	(p<0.0001)***
	GE-GA	—	—	—	(p<0.0001)***
	FIB-GA	—	—	—	—

Supplementary Table S14 Statistic test of relative correct rate (RCR) of training results.

Class	method	BP	MW	GE-GA	FIB-GA
Square	BP	—	(p=0.0005)***	(p=0.1332)ns	(p<0.0001)***
	MW	—	—	(p=0.0795)ns	(p<0.0001)***
	GE-GA	—	—	—	(p=0.5192)ns
	FIB-GA	—	—	—	—
Circle	BP	—	(p=0.9692)ns	(p=0.8471)ns	(p=0.0005)***
	MW	—	—	(p=0.8698)ns	(p=0.0431)*
	GE-GA	—	—	—	(p=0.0655)ns
	FIB-GA	—	—	—	—

Supplementary Table S15 Statistic test of relative correct rate (RCR) of one faulty parameter

Class	method	BP	MW	GE-GA	FIB-GA
Square	BP	—	(p=0.2633)ns	(p=0.0005)***	(p<0.0001)***
	MW	—	—	(p<0.0001)***	(p<0.0001)***
	GE-GA	—	—	—	(p<0.0001)***
	FIB-GA	—	—	—	—
Circle	BP	—	(p=0.5220)ns	(p<0.0001)***	(p<0.0001)***
	MW	—	—	(p<0.0001)***	(p<0.0001)***
	GE-GA	—	—	—	(p<0.0001)***
	FIB-GA	—	—	—	—

Supplementary Table S16 Statistic test of relative correct rate (RCR) of one faulty neuron

Class	method	BP	MW	GE-GA	FIB-GA
Square	BP	—	(p=0.3824)ns	(p=0.0044)**	(p<0.0001)***
	MW	—	—	(p=0.0001)***	(p<0.0001)***
	GE-GA	—	—	—	(p<0.0001)***
	FIB-GA	—	—	—	—
Circle	BP	—	(p=0.6563)ns	(p<0.0001)***	(p<0.0001)***
	MW	—	—	(p<0.0001)***	(p<0.0001)***
	GE-GA	—	—	—	(p<0.0001)***
	FIB-GA	—	—	—	—

Supplementary Table S17 Statistic test of relative correct rate (RCR) of multiple faulty parameters

Number	Class	method	BP	MW	GE-GA	FIB-GA
2	Square	BP	—	(p=0.2336)ns	(p=0.0003)***	(p<0.0001)***
		MW	—	—	(p<0.0001)***	(p<0.0001)***
		GE-GA	—	—	—	(p<0.0001)***
		FIB-GA	—	—	—	—
	Circle	BP	—	(p=0.7904)ns	(p<0.0001)***	(p<0.0001)***
		MW	—	—	(p<0.0001)***	(p<0.0001)***
		GE-GA	—	—	—	(p<0.0001)***
		FIB-GA	—	—	—	—
3	Square	BP	—	(p=0.2034)ns	(p=0.0005)***	(p<0.0001)***
		MW	—	—	(p<0.0001)***	(p<0.0001)***
		GE-GA	—	—	—	(p<0.0001)***
		FIB-GA	—	—	—	—
	Circle	BP	—	(p=0.9760)ns	(p<0.0001)***	(p<0.0001)***
		MW	—	—	(p<0.0001)***	(p<0.0001)***
		GE-GA	—	—	—	(p<0.0001)***
		FIB-GA	—	—	—	—

Supplementary Table S18 Statistic test of relative correct rate (RCR) of multiple faulty neurons

Number	Class	method	BP	MW	GE-GA	FIB-GA
2	Square	BP	—	(p=0.6388)ns	(p=0.0053)**	(p<0.0001)***
		MW	—	—	(p=0.0032)**	(p<0.0001)***
		GE-GA	—	—	—	(p<0.0001)***
		FIB-GA	—	—	—	—
	Circle	BP	—	(p=0.4373)ns	(p<0.0001)***	(p=0.0036)**
		MW	—	—	(p=0.0003)***	(p=0.0003)***
		GE-GA	—	—	—	(p<0.0001)***
		FIB-GA	—	—	—	—
3	Square	BP	—	(p=0.7594)ns	(p=0.0490)*	(p=0.0001)***
		MW	—	—	(p=0.0260)***	(p=0.0004)***
		GE-GA	—	—	—	(p<0.0001)***
		FIB-GA	—	—	—	—
	Circle	BP	—	(p=0.8382)ns	(p=0.0223)*	(p=0.3303)ns
		MW	—	—	(p=0.0106)*	(p=0.4081)ns
		GE-GA	—	—	—	(p=0.0037)**
		FIB-GA	—	—	—	—
